# The Oxidative State of Cysteine Thiol 144 Regulates the SIRT6 Glucose Homeostat

**DOI:** 10.1038/s41598-017-11388-6

**Published:** 2017-09-08

**Authors:** David Long, Hanzhi Wu, Allen W. Tsang, Leslie B. Poole, Barbara K. Yoza, Xianfeng Wang, Vidula Vachharajani, Cristina M. Furdui, Charles E. McCall

**Affiliations:** 1 Department of Internal Medicine, Section on Molecular Medicine, Wake Forest School of Medicine, Winston-Salem, NC 27157 USA; 2 Department of Biochemistry, Wake Forest School of Medicine, Winston-Salem, NC 27157 USA; 3 Department of Surgery, Wake Forest School of Medicine, Winston-Salem, NC 27157 USA; 4 Department of Anesthesiology, Section on Critical Care, Wake Forest School of Medicine, Winston-Salem, NC 27157 USA

## Abstract

Control of glucose homeostasis plays a critical role in health and lifespan and its dysregulation contributes to inflammation, cancer and aging. NAD + dependent Sirtuin 6 (SIRT6) is a glucose homeostasis regulator in animals and humans and its regulation at the molecular level is unknown. Here, we report that a cysteine thiol redox sensor contributes to the role of SIRT6 in controlling glucose homeostasis. Sulfenylation of SIRT6 occurs in THP1 cells and primary human promonocytes during inflammation and in splenocytes from mice with sepsis. Inhibiting xanthine oxidase, a major reactive oxygen species (ROS) contributor during acute inflammation, reduces sulfenylation of SIRT6, glucose transporter Glut1 expression, glucose uptake, and glycolysis. A block in glycolysis associated with monocyte deactivation by endotoxin, a process contributing to immunometabolic paralysis in human and mouse sepsis monocytes, can be reversed by increasing H_2_O_2_ and sulfenylating SIRT6. Mutation analysis of SIRT6 Cys144, which lies in its phylogenetically conserved zinc-associated Cys-X-X-Cys motif near the catalytic domain of the protein, decreases SIRT6 deacetylase activity and promotes glycolysis. These results suggest that direct and reversible cysteine thiol 144 may play a functional role in SIRT6-dependent control over monocyte glycolysis, an important determinant of effector innate immune responses.

## Introduction

The discovery of the NAD+ reacting function of the yeast Silent Information Regulator Two (Sir2) gene introduced the role of the SIRT family as redox and metabolic sensors, a concept later confirmed in humans^1^. NAD+ dependent Sirtuin 6 (SIRT6) is a glucose homeostasis regulator in animals and humans^2, 3^ and its regulation at the molecular level is unknown. Human nuclear SIRT1 and 6 epigenetically coordinate a transition between Warburg-like aerobic glycolysis and lipolysis during acute inflammation^4^, and the acute inflammatory process both generates and removes reactive oxygen and nitrogen species (RNS)^5^. ROS and RNS both injure cells and tissues during inflammation, but also instruct cell signaling by reversible cysteine oxidation (e.g., sulfenylation, disulfide formation)^6^. Sulfenylation of signaling, metabolic and epigenetic proteins have profound impact on enzymatic activity or interaction with other proteins as reviewed recently^7^.

**Figure 1:**
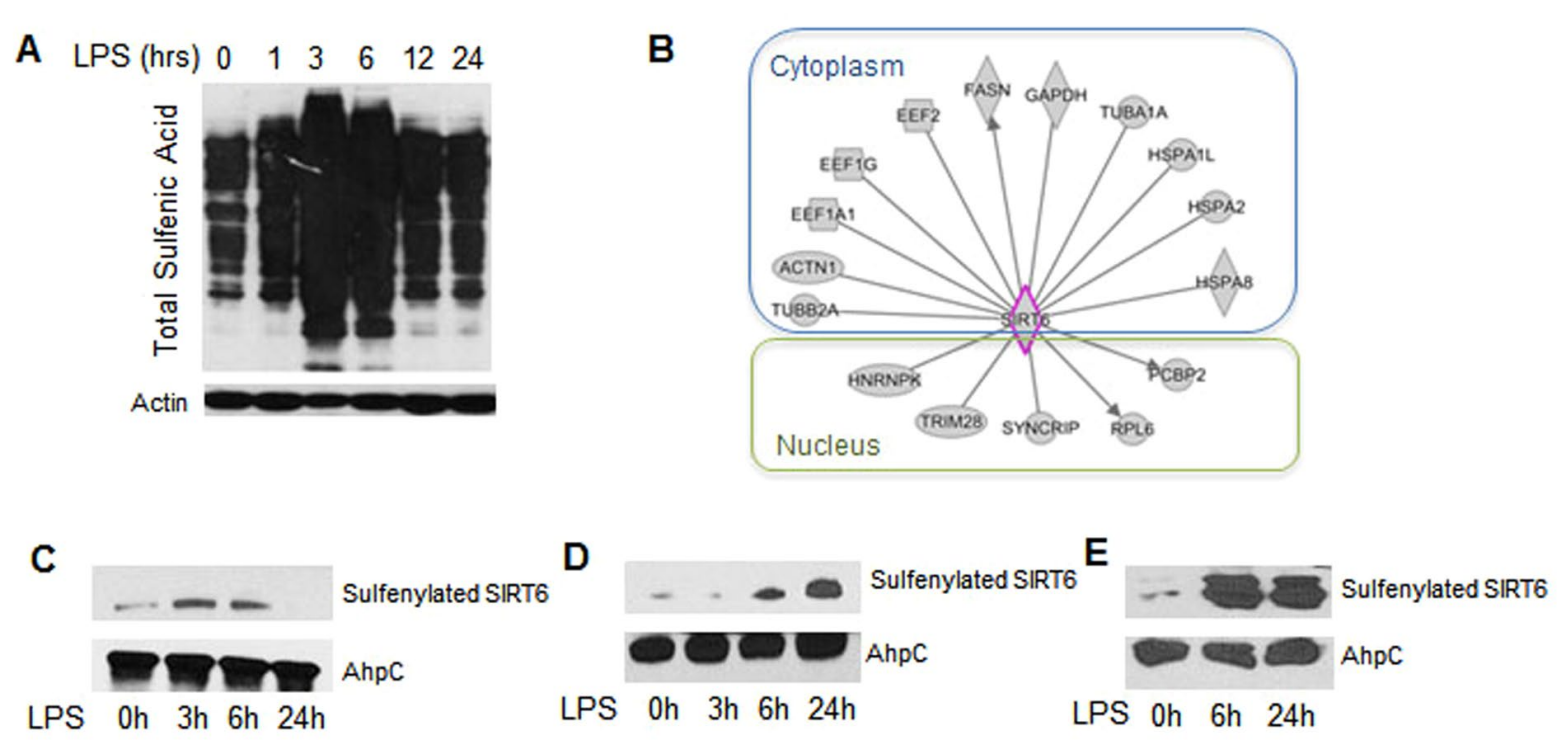
Total protein sulfenylation and SIRT6 specific sulfenylation is increased in THP-1 cells and SIRT6 sulfenylation is increased in human monocytes stimulated with LPS and in mouse spleen subjected to CLP-induced sepsis: (**A**) Effect of LPS stimulation on sulfenylation in THP-1 cells: Cells were stimulated with LPS over a time course of 0–24 hours and lysates were evaluated for total protein sulfenic acid formation (*n* = 3), (**B**) Mass spectrometry analysis identified sulfenylated proteins at 3 and 6 h time points mapping to SIRT6 interactome. (**C**) Evidence of SIRT6 sulfenylation: Cells were stimulated with LPS over a time course of 0–24 hours and lysates were evaluated for SIRT6 sulfenylation (*n* = 5). (**D**) Human adherent monocytes isolated from peripheral blood were stimulated with LPS over a time course of 0–24 hours and lysates were evaluated for SIRT6 sulfenylation (*n* = 3). (**E**) Spleen from mice subjected to CLP at 6 and 24 hours was lysed and lysates were evaluated for SIRT6 sulfenylation (n = 3).

To determine the kinetics of direct protein cysteine thiol oxidation during the acute inflammatory response of human monocytes, we used a selective biotin tagged sulfenic acid probe BP1 (1,3-cyclopentanedione (BP1)^8, 9^ to first model sulfenylation kinetics following lipopolysaccharide (LPS) stimulation in human THP1 promonocyte cultures. Since acute inflammation transitions between proinflammatory initiation and anti-inflammatory adaptation states^10^, we stimulated cultured cells with 1 μg/mL LPS between 1–24 h and lysed them in the presence of the BP1 detection reagent. Immunoblotting with an anti-biotin antibody identified multiple sulfenylated proteins, with protein sulfenic acid peaking at 3–6 hours and returning to baseline levels at 24 h (Fig. 1A). Mass spectrometry analysis of BP1-labeled proteins at 3 and 6 h time point of LPS stimulation distinguished 133 proteins. Ingenuity Pathway Analysis (IPA) defined glycolysis as one of the top pathways represented in the data with 7 proteins mapping to this pathway: aldolase a and c, enolase 1, 2, and 3, glyceraldehyde phosphate dehydrogenase, and phosphoglycerate kinase-1.

A recent report supported that SIRT6 sulfenylation of recombinant protein *in vitro* alters its binding to HIF1α glycolysis regulator^11^. SIRT6 is a known master regulator of glucose metabolism *in vivo* during a time in which ROS signaling regulates acute inflammation^4^, and dysregulated glycolysis and ROS generation have been linked to the pathologic role of monocytes in obesity, diabetes, and chronic inflammation^12, 13^. Thus, we hypothesized that SIRT6 sulfenylation, although not detected by mass spectroscopy analysis of protein sulfenylation in our study of total proteins, is a likely candidate for *in vivo* sulfenylation. First, and to determine if the protein sulfenylation dataset includes potential SIRT6 binding proteins, we overlaid the dataset on the SIRT6 interactome containing 217 experimentally validated direct interactions (protein binding, activation of expression, and others)^2, 14–16^. The analysis identified 17 proteins sulfenylated in response to LPS as potential SIRT6 interacting, proteins including the glycolysis enzyme GAPDH and lipid metabolism enzyme fatty acid synthase FASN, the expression of which is regulated by SIRT6 (Fig. 1B). Also, SIRT6 deacylases activity is markedly increased by its binding to fatty acids^17^. These findings suggested that SIRT6 might be regulated by sulfenylation.

We next used streptavidin to isolate biotinylated proteins from BP1 labeled samples from THP1 monocytes followed by immunoblotting with SIRT6 antibody to detect sulfenylation. SIRT6 was sulfenylated at 3 and 6 h, with a return to baseline at 24 h (Fig. 1C). We utilized two different models validate this observation of SIRT6 sulfenylation: normal monocytes isolated from peripheral human blood and stimulated with LPS, and mouse splenocytes isolated from mice subjected to cecal ligation and puncture (CLP) to induce acute systemic infection from sepsis. In mouse sepsis, as well as human sepsis monocytes, we reported that elevated aerobic Warburg glycolysis of the early anabolic state of acute inflammation depends on both nuclear SIRT1 and 6 to transition to a fatty acid oxidation dominant catabolic state, which during septic shock becomes immunosuppressive, limits organ function and is often lethal^10^. Human primary monocytes assessed *ex vivo* showed increased sulfenylation of SIRT6 when stimulated with LPS (Fig. 1D). Mouse splenocytes isolated during the acute inflammatory state of sepsis induced by CLP, which have elevated glycolysis^4, 18^ and ROS generation^19^, also showed increased sulfenylation of SIRT6 (Fig. 1E). In both primary monocytes and splenocytes from septic mice, increases in SIRT6 sulfenylation persisted longer than the cell culture model.

**Figure 2:**
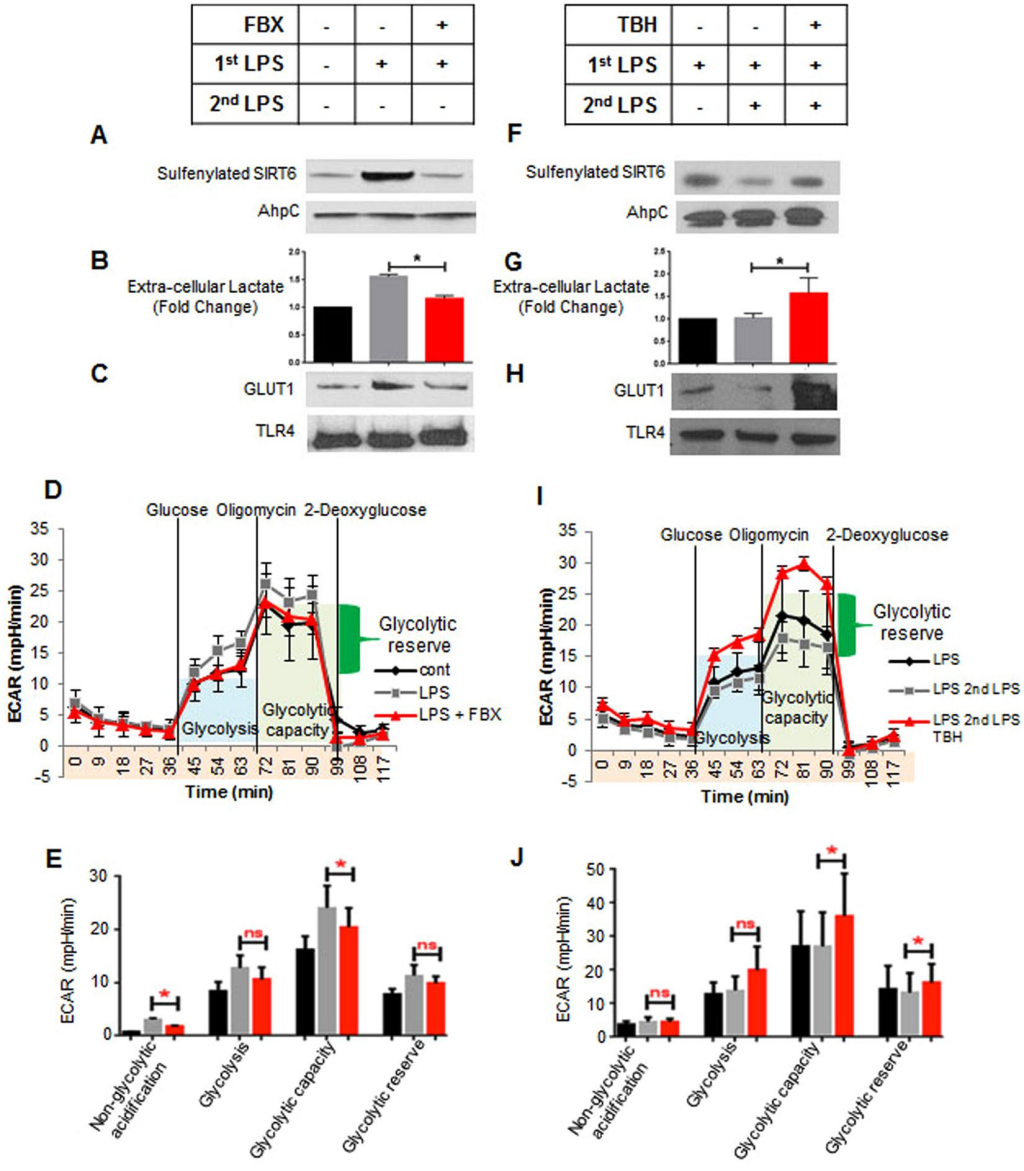
Antioxidant treatment prevents LPS induced SIRT6 sulfenylation and glycolysis in LPS sensitive THP-1 cells, whereas prooxidant treatment enhances LPS induced SIRT6 sulfenylation and glycolysis in LPS tolerant THP-1 cells: (**A–E)**, Effect of Febuxostat (FBX) treatment on Glycolysis in sensitive cells: (**A**) Cells were pretreated 30 minutes with 30 µM FBX followed by 3 hour LPS stimulation. Lysates were made and evaluated for SIRT6 sulfenylation (*n* = 3), (**B**) Cells were pretreated 30 minutes with 30 µM FBX followed by 6 hour LPS stimulation. Media was collected and evaluated for lactate production (*n* = 6), (**C**) Cells were pretreated 30 minutes with 30 µM FBX followed by 4.5 hour LPS stimulation. Membrane fractions were made and evaluated for Glut-1 expression (*n* = 3). (**D**,**E**) Cells were pretreated 30 minutes with 30 µM FBX followed by 6 hour LPS stimulation. Cells were evaluated for Glycolysis by Seahorse Respirometry (*n* = 3) (**F–J**), Effect of Tert-butyl hydroperoxide (TBH) treatment on Glycolysis in tolerant cells: (**F**) Tolerant cells were pretreated 30 minutes with 120 µM TBH followed by 3 hour LPS stimulation. Lysates were made and evaluated for SIRT6 sulfenylation (*n* = 3), (**G**) Tolerant cells were pretreated 30 minutes with 120 µM TBH followed by 6 hour LPS stimulation. Media was collected and evaluated for lactate production (*n* = 4), (H) Tolerant cells were pretreated 30 minutes with 120 µM TBH followed by 4.5 hour LPS stimulation. Membrane fractions were made and evaluated for Glut-1 expression (*n* = 3). *P < 0.05 (two-tailed Student’s *t*-test for pairwise comparisons), (**I–J**) tolerant cells were pretreated 30 minutes with 120 µM TBH followed by 6 hour LPS stimulation. Cells were evaluated for glycolysis by Seahorse Respirometry (n = 3).

Xanthine oxidase (XO) is a form of purine oxidoreductase, which catalyzes the oxidation of hypoxanthine derived from adenine to xanthine and uric acid, is a major ROS generator in LPS-stimulated THP-1 monocytes^20^. Since SIRT6 was sulfenylated during LPS stimulation, we next determined the effect of XO-dependent ROS inhibition on SIRT6 sulfenylation. Pretreatment of THP1 cells with febuxostat, an inhibitor of xanthine oxidase, significantly decreased LPS-induced SIRT6 sulfenylation (Fig. 2A). We then pretreated THP1 cells with febuxostat to inhibit ROS prior to LPS stimulation and examined several parameters related to glycolysis. Pretreatment with febuxostat inhibited LPS induced extracellular lactate production (Fig. 2B). We next subjected cells to a glycolysis stress test using a Seahorse XF^e^ Extracellular Flux Analyzer to assess glucose import, glycolysis, and glucose metabolic capacity. Pretreatment with febuxostat decreased LPS induced glucose intake, glycolysis, and glycolytic capacity (Fig. 2D and E). We then examined membrane expression of Glucose Transporter GLUT1, which has increased expression during the early acute inflammatory response of many immune cells and is required for optimal host defense against infection^21^. Pretreatment with febuxostat inhibited GLUT1 membrane expression (Fig. 2C). Taken together these results suggest that ROS play a vital role in LPS-induced glycolysis functions including glucose uptake and that oxidation and reduction might control SIRT6 as a glucose management homeostat.

A recent report showed that human monocytes assessed *ex vivo* during severe systemic inflammation from sepsis are unable to mount a second glycolysis burst and lactate generation when rechallenged with a second dose of LPS^22^; paralyzed glycolysis to endotoxin stimulation (endotoxin desensitization/tolerance) also occurred *in vitro* in monocyte models of acute sepsis-induced inflammation in mice and human blood monocytes obtained from septic patients. That study also reported that monocytes with deactivated glycolysis also were unable to increase oxidative phosphorylation and ROS generation in response to a second LPS stimulation. Having demonstrated that LPS induced SIRT6 oxidation and glycolysis was dependent on ROS production, we determined the effects of ROS treatment on SIRT6 oxidation and glycolysis in endotoxin tolerant cells, which are unable to mount a glycolysis response. To desensitize cells to endotoxin, we stimulated them with 1 µg/mL LPS. The next day cells were washed and replated prior to subsequent treatments and re-stimulation with LPS. To test ROS effects, we treated endotoxin tolerant cells with tert-butyl hydroperoxide (TBH), a hydrogen peroxide mimetic that induces protein sulfenylation^23^, prior to restimulating with LPS. LPS treatment did not increase SIRT6 sulfenylation in endotoxin tolerant cells, but SIRT6 was sulfenylated when cells were pretreated with TBH prior to restimulation (Fig. 2F). Additionally, LPS treatment did not increase extracellular lactate production or glycolytic function in tolerant cells, but when cells were treated with TBH prior to the second LPS stimulation extracellular lactate accumulation, glycolysis, and GLUT1 expression was restored (Fig. 2G–J). Not shown is that the antimalarial drug, Artemisinin^24^, which is an organic hydroperoxide, inactivates SIRT6 control over glycolysis. These data support that impaired glycolysis in endotoxin-deactivated monocytes reverses when cells shift from a reduced to oxidized environment, an effect that could be beneficial in an immune repressed state seen in inflammation and cancer.

**Figure 3:**
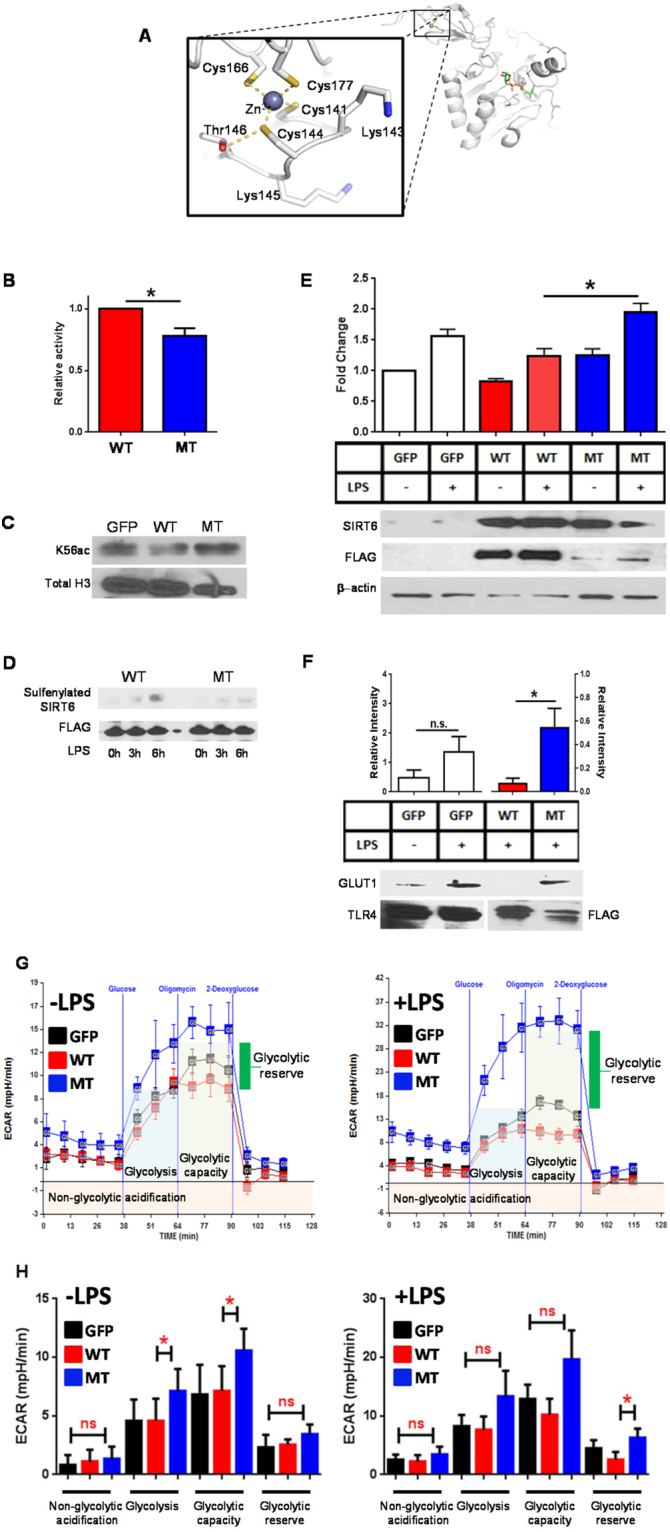
SIRT6 Cys144 displays lower activity than wild type and promotes pro-inflammatory glycolysis. (**A**) Cysteine residues coordinating the Zn-motif in Sirt6 are shown based on the 3PKI Sirt6 structure extracted from the Protein Data Bank. (**B**) SIRT6 activity assay was performed on cells transfected with wild-type SIRT6 plasmid or Cys144 mutant SIRT6 plasmid (*n* = 3). (**C**) Histone preparations were made on cells transfected with wild-type SIRT6 plasmid or Cys144 mutant SIRT6 plasmid and preparations were immunoblotted with H3K56 antibody (*n* = 3). (**D**) Cells transfected with wild-type SIRT6 or Cys144 mutant SIRT6 were stimulated with LPS for 3 and 6 hours and subjected to SIRT6 sulfenylation analysis (n = 3). (**E**–**H**) Effect of Cys144 mutation on Glycolysis in THP-1 cells: (**E**) Cells transfected with wild-type SIRT6 or Cys144 mutant SIRT6 were stimulated with LPS overnight. Media was collected for analysis of extracellular lactate production (*n* = 5). (**F**) Cells transfected with wild-type SIRT6 or Cys144 mutant SIRT6 were stimulated with LPS for 4.5 hours. Membrane fractions were made and evaluated for Glut-1 expression (*n* = 3). *P < 0.05 (two-tailed Student’s *t*-test for pairwise comparisons). (**G**,**H**) Cells transfected with wild-type SIRT6 or Cys144 mutant SIRT6 were stimulated with LPS for 6 hours and Glycolysis was assessed by Seahorse Respirometry (*n* = 4).

All SIRT family members share a common Cys–X_2_–Cys–X_15–20_–Cys–X_2_–Cys amino acid motif that is responsible for Zinc cofactor binding^25^. Although the Zinc region is not in the SIRT catalytic site, the cysteines in the motif can be oxidized to alter SIRT function; such as reported for nitrosylation and deactivation of SIRT1 deacetylase^26^. We speculated that this motif in SIRT6 would be important for control of its function during acute inflammation in the reprogramming of immunity to an immunosuppressive state during prolonged sepsis (Fig. 3A). We therefore constructed a FLAG-tagged SIRT6 with cysteine to serine mutation at position 144, which is the second cysteine of the Zinc binding motif. This plasmid as well as a FLAG-tagged wild type (WT) SIRT6 plasmid, were transfected into THP-1 cells. In our first experiment with the WT and mutant SIRT6 construct, we determined if the mutation would affect SIRT6 activity. To do this, overexpressed WT and Cys144Ser SIRT6 were immunoprecipitated with anti-FLAG beads and a SIRT6 enzymatic deacetylation activity assay was performed on immunoprecipitated beads. The Cys144Ser mutant showed modest but significant decreases in activity relative to WT SIRT6 (Fig. 3B). Histone H3K56 is a known histone deacetylation target of SIRT6, which supports DNA repair^27^. To test the effect of Cys144Ser SIRT6 mutation on histone acetylation within cells, we transfected control, WT SIRT6, and Cys144Ser SIRT6 plasmids into THP-1 cells, isolated the histone fraction, and immunoblotted histone with anti-H3K56 acetylation antibody. WT SIRT6 transfected cells decreased acetylation of H3K56, suggesting that its deacetylase was activated. In contrast, Cys144Ser mutant transfected cells did not decrease acetylation of H3K56, suggesting that the deacetylase was not active (Fig. 3C). Together these two pieces of data support that the Cys144 site regulates SIRT6 activity and may impact glucose homeostasis. To develop this model, we first tested whether Cys144 mutation altered sulfenylation of SIRT6. To optimize this, a FLAG antibody pull-down was performed on BP-1 sulfenic acid labeled lysates from both WT and Cys144Ser SIRT6 transfected samples. Immunoblotting with anti-biotin antibody revealed that in the WT transfected cells stimulated with LPS, SIRT6 has increased sulfenylation while increases in sulfenylation were not appreciable in Cys144Ser SIRT6 transfected cells stimulated with LPS (Fig. 3D). These results support that Cys144 is a functional site for informing SIRT6 effects on glucose management in monocytes.

Since SIRT6 is a major regulator of glycolysis and glucose homeostasis, we examined the effect of Cys144 mutation on glycolysis by transfecting with WT and Cys144 mutant SIRT6 or control plasmid and stimulated cells with LPS, which elevates glycolysis. We used 3 ways to assess glucose metabolism under these conditions: lactate accumulation by biochemical analysis, GLUT1 glucose transporter expression, glycolysis by Seahorse glucose stress test. We found that WT construct transfected cells produced slight decreases in extracellular lactate (Fig. 3E), glycolysis (Fig. 3G,H), and GLUT-1 expression (Fig. 3F) over control transfected cells after LPS stimulation. In contrast, cells transfected with Cys144Ser mutant significantly increased extracellular lactate (Fig. 3E), glycolytic capacity (Fig. 3G,H), but did not decrease GLUT-1 expression (Fig. 3F). These results were obtained even though SIRT6 is constitutively expressed in THP1 monocytes. These results support that Cys144 of SIRT6 is a functional redox sensitive site for regulating glucose metabolism in monocytes.

An extended potential of this study is that the conserved Zinc cysteine motif may play a functional role in regulating the enzymatic properties of other or all members of the SIRT family and may be a part of a functional specific cysteine network. Mechanistically, diffusion of H_2_O_2_ or location-specific ROS or RNS might integrate metabolic sensing and the homeostasis protection features of this family of important stress sensors. For example, SIRT1 induces SIRT6 in monocytes and both are deactivated by sulfenylation^26, 28^. Other important questions raised by this study are whether and what reductive processes might reverse oxidation and retrieve SIRT6-dependent glycolysis homeostasis. Another important unanswered question is whether SIRT6 functional Cys144 might inform DNA repair processes in inflamed or neoplastic cells as suggested by our findings of H3K56 acetylation is altered by redox. In any event, the results of this and other studies support the possibility that a protein network containing functional cysteine thiol sites guards homeostasis and control inflammation, neoplasia, and aging.

## Methods

### Human Promonocytic THP-1 Cell Model

Promonocyte THP-1 cells from the American Type Culture Collection (ATCC) were maintained in complete RPMI 1640 medium (Invitrogen) supplemented with 100 units/ml of penicillin, 100 μg/ml of streptomycin, 2 mM L-glutamine, and 10% fetal bovine serum in a humidified incubator with 5% CO_2_ at 37 °C. Acute inflammatory response was induced by stimulating THP-1 cells with 1 μg/ml of Gram-negative bacteria lipopolysaccharide (LPS, Escherichia coli serotype 0111:B4, Sigma) for the indicated times.

### Protein Sulfenic Acid Detection

Lysates for evaluation of both global and protein-specific cysteine sulfenic acid levels were prepared using lysis buffer supplemented with 200 units/mL of catalase (from bovine liver; Sigma) and 1 mM Biotin-1,3-cyclopentanedione (BP1), a biotin tagged dimedone derivative (Kerafast). Samples were rotated end-over-end at 4 °C for 30 minutes and then 20 mM iodoacetamide (Thermo Fisher) was added to block free sulfhydryls. Samples were rotated for an additional 30 minutes at 4 °C and then centrifuged at 15,000 × g for 10 minutes at 4 °C to remove the insoluble fraction.

### Western blot analysis

For detection of total Protein Sulfenic Acid content, lysates labeled with BP1 were probed with HRP-linked anti Biotin antibody, followed by actin immunoblotting to confirm equal protein labeling. For detection of SIRT6 sulfenylation, equal amounts of protein from BP-1 labeled lysate were isolated with Streptavidin beads. Beads were washed with 1 M NaCl, 2 M Urea, 0.1% SDS/10 mM DTT, and PBS. Washed beads were subjected to SDS-PAGE and subsequent immunoblotting with SIRT6 specific antibody. During streptavidin isolation, lysates were spiked with 100 nanograms recombinant biotinylated AhpC protein per 100 micrograms protein. Immunoblotting of pull-down fractions with anti AhpC antibody was used as an internal control for streptavidin isolation efficiency.

For detection of SIRT6 sulfenylation in transfected cells, BP-1 labeled lysates were isolated with anti-FLAG magnetic beads. Beads were washed as above and were subjected to SDS-PAGE and subsequent immunoblotting with HRP-linked antibody. Immunoblotting with anti-FLAG antibody confirmed equal isolation of transfected proteins.

### Mass spectrometry analysis

Lysates for mass spectrometry analysis were prepared using lysis buffer supplemented with 200 units/mL of catalase (from bovine liver; Sigma), 1 mM Biotin-1,3-cyclopentanedione (BP1, a biotin tagged dimedone derivative), and 10 mM 4-(5-Methanesulfonyl-[1,2,3,4]tetrazol-1-yl)-phenol (MSTP, a thiol-blocker) Samples were rotated end-over-end at 4 °C for 30 minutes, centrifuged at 15,000 × g for 10 minutes at 4 °C to remove the insoluble fraction, and acetone precipitated to remove the unreacted BP1. The protein pellet was resolubilized using 0.1% SDS in PBS, the 3 and 6 h lysates were combined 1:1 based on protein concentration (500 μg protein), and enriched using streptavidin beads. The beads were washed sequentially with 2 M urea, 1 M NaCl, 0.1% SDS, and 10 mM DTT in pH 7.5 HEPES buffer, followed by the final wash with 50 mM ammonium bicarbonate. Trypsin digestion was performed on beads and the resulting peptides were acidified to 1% formic acid prior to centrifugation at 2,000 g for 5 min to remove any precipitate. The mass spectrometry analysis was performed using a Thermo LTQ Orbitrap Velos Pro high-resolution mass spectrometer interfaced with an Ultimate3000 nanoLC system (Thermo Fisher, Waltham, MA, USA/Dionex, Sunnyvale, CA, USA). Raw data files were searched against the human proteome database using the Mascot search engine and Proteome Discoverer v. 1.4 (Thermo Fisher).

### Membrane Protein Preparation

Membrane fractions were prepared using the MemPer Plus Extraction Kit (Thermo Scientific). Equal amounts of protein from each membrane fraction were subjected to SDS-PAGE and were immunoblotted with anti GLUT1 antibody (Genetex), followed by immunoblotting with anti TLR4 antibody (Santa Cruz) and/or anti-FLAG antibody. Blots were scanned and densitometry analysis was performed using ImageJ software. GLUT1 protein levels in GFP samples are expressed relative to TLR4; GLUT1 protein levels in WT and MT samples are expressed relative to FLAG.

### Histone Preparations

Histone preparations were made using the EpiQuick Total Histone Exraction Kit (EpiGentek). Equal amounts of protein from each histone fraction were subjected to SDS-PAGE and were immunoblotted with anti H3K56 acetyl specific antibody (Active Motif), followed by immunoblotting with total H3 antibody (Active Motif) to confirm equal amount of histone protein loading.

### Plasmid Construction and Transfection

Plasmid pcDNA3.1 containing an insert to express human SIRT6 (Addgene 13817) was used as the template for site-directed mutagenesis. Cysteine at position 144 was mutated to serine by introducing a single base exchange using the QuikChange site-directed mutagenesis kit. Mutants were confirmed by DNA sequencing. Plasmids were isolated using Endofree plasmid purification Kit (Qiagen).

For some experiments THP-1 cells were transfected with 1 microgram plasmid using either GeneX Plus transfection reagent (American Type Culture Collection) or Viromer Red transfection reagent (Lipocalyx) according to manufacturer’s instructions. For other experiments THP-1 cells were transfected with 500 nanograms plasmid using CellLine V nucleofector kit (Amaxa) according to manufacturer’s instructions. pMax GFP plasmid (Lonza) was used as a control plasmid to visualize transfection efficiency. Overexpression of SIRT6 protein was confirmed by immunoblotting transfected cell lysates with anti-FLAG tag antibody (Cell Signaling).

### Measurement of Glycolytic Function

Glycolytic Function was assessed using the Seahorse SF Glycolysis Stress test. Following treatment 2 × 10^5^ THP-1 cells were washed twice with glucose free media and plated into a Cell-Tak coated plate as described previously^7^. Cells were preincubated for 1 h at 37 °C in a CO_2_-free incubator. Cells were then loaded into the analyzer and extracellular acidification rate (ECAR) was measured at baseline and then following sequential injections of Glucose (10 mM), Oligomycin (1uM), and 2-Deoxyglucose (50 mM). ECAR values for non-glycolytic acidification, glycolysis, glycolytic capacity and glycolytic reserve (glycolytic capacity – glycolysis) were calculated for >3 separate observations. 1-way ANOVA with Sidak’s multiple comparisons test was performed on each physiological parameter measured within each of the separate experiments to determine significance.

### Measurement of Extracellular Lactate

Following treatment 1 × 10^5^ THP-1 cells were collected from each condition and diluted to a final volume of 200 microliters with cell culture media. Cells were spun down and media was collected. 5 microliters of media from each sample in duplicate were analyzed for the presence of lactate using the Glycolysis Cell Based Assay Kit (Cayman Chemical) according to manufacturer’s instructions.

### SIRT6 activity

48 hours after transfection cells were collected and sonicated in ice-cold RIPA Lysis and Extraction Buffer. The lysates were centrifuged at 13800 g for 5 minutes at 4 °C. Supernatants were collected and protein concentrations were determined by Coomassie Plus Assay (Fisher Scientific). Samples were stored at −80 °C until used. An aliquot corresponding to 1400 micrograms was taken from each lysate and lysates were immunoprecipitated overnight with anti-FLAG magnetic beads (Sigma Aldrich). SIRT6 activity was then performed using SIRT6 Inhibitor Screening Kit (Biovision) according to the following protocol: Beads were washed three times with PBS and three times with SIRT6 activity assay buffer. Washed beads were resuspended in SIRT6 Activity Assay Buffer. Substrate was added to beads and samples were incubated for 1 hour at 37 °C. Developer was then added to beads and samples were incubated for 10 minutes at 37 °C. Supernatant was collected from beads and was transferred into two separate wells of a black 96 well plate. The fluorescence intensity was measured using a microtiter plate fluorometer with excitation at 400 nm and emission at 500 nm. Protein was eluted from beads by boiling in sample buffer for 10 minutes. Boiled supernatant was loaded onto gel and immunoblotted for FLAG. Blots were scanned and densitometry analysis was performed using ImageJ software. SIRT6 activity was normalized to the quantitation of FLAG by immunoblot for each experiment.

### Human Lymphocyte and Murine Splenocyte Studies

All study methods were performed in accordance with protocols approved by the institution in accordance with federal and state guidelines. Primary lymphocytes were collected from de-identified heparinized venous blood samples obtained with written informed consent from healthy adult volunteers according to the Institutional Review Board protocol approved by Wake Forest University. RBCs, platelets, and polymorphonuclear neutrophils were removed through Isolymph (Gallard-Schlesinger Industries) centrifugation of whole blood. Monocytes were then enriched through a 2-h adherence step, after which nonadherent cells were removed. Cells were then cultured overnight in fresh RPMI 1640 containing 10% FBS prior to stimulation with 100ng/mL LPS for the indicated times. Cells were lysed with BP-1 lysis buffer as above.

The sepsis model of cecal ligation and puncture (CLP) was generated with C57BL/6 (6–8-week-old) mice according to a protocol approved by the Wake Forest University School of Medicine Institutional Animal Care and Use Committee. All mice were obtained from Jackson Laboratories (Bar Harbor, ME), and CLP procedure (two punctures with 22-gauge needles) was performed under anesthesia as described^29^.

Control, 6 h and 24 h-CLP mice were anesthetized with ketamine (150 mg/kg intramuscular) and xylazine (7.5 mg/kg intramuscular). Each mouse then underwent jugular venous and carotid arterial cannulation. BP-1 was injected intravenously (25 mg/kg in 200 microliter normal saline) and allowed to circulate for 30 minutes. Isovolemic blood exchange was performed to remove all unbound BP-1 from the circulation. Follwing BP-1 labeling organs were harvested from mice and snap frozen in liquid nitrogen. Snap frozen spleens were then lysed by homogenization in RIPA lysis buffer. Spleen lysates were then subjected to SIRT6 sulfenylation analysis as above.
